# Federated Learning in the Detection of Fake News Using Deep Learning as a Basic Method

**DOI:** 10.3390/s24113590

**Published:** 2024-06-02

**Authors:** Kristína Machová, Marián Mach, Viliam Balara

**Affiliations:** Department of Cybernetics and Artificial Intelligence, Faculty of Electrical Engineering and Informatics, Technical University of Košice, Letná 9, 04200 Košice, Slovakia; marian.mach@tuke.sk (M.M.); viliam.balara@tuke.sk (V.B.)

**Keywords:** federated learning, deep learning, fake news detection, natural language processing

## Abstract

This article explores the possibilities for federated learning with a deep learning method as a basic approach to train detection models for fake news recognition. Federated learning is the key issue in this research because this kind of learning makes machine learning more secure by training models on decentralized data at decentralized places, for example, at different IoT edges. The data are not transformed between decentralized places, which means that personally identifiable data are not shared. This could increase the security of data from sensors in intelligent houses and medical devices or data from various resources in online spaces. Each station edge could train a model separately on data obtained from its sensors and on data extracted from different sources. Consequently, the models trained on local data on local clients are aggregated at the central ending point. We have designed three different architectures for deep learning as a basis for use within federated learning. The detection models were based on embeddings, CNNs (convolutional neural networks), and LSTM (long short-term memory). The best results were achieved using more LSTM layers (F1 = 0.92). On the other hand, all three architectures achieved similar results. We also analyzed results obtained using federated learning and without it. As a result of the analysis, it was found that the use of federated learning, in which data were decomposed and divided into smaller local datasets, does not significantly reduce the accuracy of the models.

## 1. Introduction

Nowadays, the amount of information on the web and its availability are growing, and these are accompanied by an increase in misinformation, especially in the form of fake news. The pandemic era associated with COVID-19 has reinforced these trends, as people have moved more widely into the online space. This misinformation is becoming more damaging to users’ health and sometimes leads to avoidable deaths.

As the amount of fake news on the internet grows, it is becoming increasingly difficult to distinguish it from true information. The problem of the dissemination of fake news is a longstanding one, but with social media it is becoming even more dangerous as fake news is spreading faster. Fake news now has serious consequences, including widespread mistrust, the manipulation of public opinion, and even threats to human health and safety.

Our research designs a fake news detection approach based on deep learning models on data about COVID-19 that would be helpful for web users to recognize fake news and, thus, not be influenced by it.

The novelty of our research is in using federated learning above models of neural networks. The security of the data from sensors at each station edge, in intelligent houses, for example, could be increased by training models separately on local data obtained from sensors in local sources—intelligent houses. Federated learning makes machine learning more secure by training models on decentralized data at decentralized edges, right where the sensor data are collected, either on some production lines or in some smart homes. If the learned model is a neural network, then it is possible to compare models learned at different edges or sources and to analyze what the different models have in common and how they differ. These differences will help us to understand the specific conditions of each source edge and, thus, contribute to the explainability of neural network models, which are still considered as black boxes.

The contributions of this article can be summarized in the following points:

- The design of three different neural network topologies, based on CNN networks, by combining a CNN and LSTM with multiple LSTM layers;
- Federated learning implementation;
- The application of federated learning to three local datasets, using all three designed architectures of the neural network;
- A comparison of test results obtained using federated learning and without federated learning;
- Datasets with fake news about COVID-19. The datasets “fake_news.csv” and “real_news.csv” are available at https://kristina.machova.website.tuke.sk/useful/ (accessed on 22 March 2024).

### 1.1. Fake News

Everyone has probably been confronted with the term “fake news”. It is misleading or untrue information. In most cases, fake news is presented as a tool of promotion and manipulation that can be used to achieve some kind of benefit, for example, political or economic. The study [1] analyzed articles that use fake news (FN) and defined six types of FN: satire FN, parody FN, manipulation, propaganda, fabrication, and advertising.

A common feature of these types of news is a certain ability to mimic the appearance of real, true news in the media. In this way, the credibility and legitimacy of such fake news grows, and it damages the credibility and real meaning of the media and journalism. There are four main components of fake news [2]:

- FN creators and disseminators—these can be human or even programmed artificial intelligence troll-bots. However, humans themselves are the main disseminators of fake news. The content of such FN is difficult to distinguish from true news based on content or language analysis alone;
- FN content—the content of a message has two parts, namely the physical content (e.g., the title and text of the message) and the non-physical content (e.g., the subject or purpose). Simply put, the physical content is the format and medium of the misleading news, and the non-physical content may be thoughts and opinions of the truth authors. The creators of fake news try to evoke a strong negative or positive feeling, where they also use polarization;
- Targeted victims—depending on the specific FN and content, these are usually internet users on social networking sites;
- Social context—represents the online space where the fake news is spread.

A more recent form of FN is so-called deepfake [3], which was created by combining the words “deep learning” and “fake”. It is the creation of a text, photo, or video in which, through deep learning, the face of one person is substituted for another person in the recorded video. It is also possible to falsify a model of a voice in this way. This technology can be very dangerous, indeed, and represents a rather large threat that society will certainly have to address. Another way is to focus on the detection of authors of FN. A potential intervention that is commonly used is highlighting or labeling the authors or sources of news articles as credible or, conversely, as those who provide or share FN and misleading information [4].

A very common intervention, especially in social media, is automated message detection through machine learning models, natural language processing, and social network analysis (SNA). Contributions that are evaluated as suspicious are downgraded using a rating algorithm so these posts are less likely to be available to users. However, the current problem is mainly that such misinformation content and fake news can evolve and spread quite quickly, and therefore, fake news detection models trained today probably will not be effective tomorrow, such as FN about COVID-19, for example.

We focused only on FN about COVID-19. The victims of such FN were not only people with tendencies towards conspiracy theories. For example, 71% of the US adult population had registered that the pandemic was orchestrated by a group of influential people and 20% considered it to be true [5]. Another well-known FN concerned a microchip that was said to be in the COVID-19 vaccine and could enter the body and manipulate human DNA. There was also a conspiracy theory associated with the coronavirus stating that the coronavirus was spread via 5G towers. Drugs such as ivermectin, whose efficacy against COVID-19 has never been proven, have also been discussed [6]. These false reports also led to the prolongation of the pandemic.

### 1.2. Related Works

For fake news detection, similarly to conversational content analysis in general, the following approaches can be effectively used: machine learning, sentiment analysis, or the combination of both. The approaches can be used for two different tasks—recognition of FN or of its authors. The work [7] uses machine learning and sentiment analysis as parallel approaches to identify not only fake news but its authors as well. In the work, the F1-measure with a value of 0.98 was achieved using support vector machines methods. The work [8] focuses on the detection of unreliable authors of online posts using a CNN as well as LSTM. The authors used data from Twitter and also used classical methods of machine learning such as SVM and KNN. The neural network approach achieved an accuracy = 0.93. This result was a few percentage points better in comparison with the SVM and 10% better than the KNN, methods which were also tested. Another example of using a combination of machine learning and sentiment analysis is the study [9].

Recent research published in [10] focused on evaluating machine learning methods in terms of their suitability for detecting fake news. It investigated and comprehensively evaluated several algorithms for FN detection:

1. Regression-based algorithms (L1 regularized logistic regression);
2. Algorithms based on neural networks (multi-layer perceptron (MLP) and convolutional neural networks);
3. Decision tree methods (decision trees and random forests);
4. Bayesian methods (Gaussian naive Bayes and multinomial naive Bayes);
5. C-support vector classification.

Convolutional neural networks were evaluated as the best option among the other algorithms, despite their significantly longer training time and their requirement for larger datasets.

In another paper [11], the authors discuss the design and creation of an artifact using a stacking approach to the recognition of fake news in online spaces. They experimented with algorithms such as logistic regression, support vector machine (SVM), k-NN, decision trees, random forest, convolutional neural networks, gated recurrent networks, and long short-term memory. In the experiment, they used two datasets, namely ISOT and KDnugget. Further, the paper used McNemar’s test for comparison of the models. Using the stacking approach, the two best models achieved an accuracy of 0.99 (random forest, ISOT) and 0.96 (logistic regression, KDnugget).

Some works such as [12] have further investigated fake news detection methodology which not only considers information about the content of the news but also takes additional information in regard to the use of social networks into scope. Tensor factorization was used for the generation of news representation. A comparative analysis of three approaches was presented: the first one based on news texts, the second based on the way of using social networks, and the last one based on their combination. The combined content and context approach was found to provide better results. The use of a deep neural network provided further improvement in terms of accuracy, success rate, and recall compared to the XGBoost classifier.

In another study [13], a model architecture, namely a new graph neural network model, was designed, named FakeDetector. In the work, the authors combined explicit and implicit attributes extracted from news texts for the detection of FN. FakeDetector is a deep diffusion neural network that represents and evaluates the news, authors, and the subject of an article simultaneously. Thus, the work’s goal was to train a prediction model that could also indicate authors’ credibility. In the paper, a new diffusion unit model, namely GDU, was presented. The model is a neural network based on the principle of information propagation by diffusion. Diffusion in this case means that information is spread from a neighboring neuron to an actual neuron through a diffusion process. The GDU model can process multiple inputs simultaneously and can combine inputs efficiently to generate outputs. Extensive experiments have been conducted on FN datasets. The experiments showed satisfactory levels of efficiency of the proposed approach in the detection of fake news and their authors in the online space.

In [14], a novel approach was proposed—DocEmb—to detect fake messages using text data embedding. The paper presents experiments with a variety of text representations such as TF-IDF (term frequency-inverse document frequency) or using word and transformer embedding: Word2Vec SG and CBOW, FastText SG and CBOW, GloVe, BERT, BART, and RoBERTa. The authors trained models on these text representations using machine learning methods such as naive Bayes, gradient boosted trees, and deep learning models—Perceptron, Multi-Layer Perceptron, [Bi]LSTM, and [Bi]GRU. One important result was that simple machine learning algorithms applied to pre-trained models using the proposed DocEmb approach achieved comparable results to the models that were specifically trained for FN detection using deep learning. They also concluded that the representation of words in the document plays a more important role for achieving the highest accuracy than the complexity of the model.

The majority of attempts to detect fake news usually focus only on textual information. Multimodal approaches are less common and usually classify messages as true or false. In the work [15], a multimodal fake news detection approach was described. The detection was performed using both unimodal and multimodal approaches. The results of the work showed that a multimodal approach achieved reasonably satisfactory results in the case where it was based on a convolutional neural network (CNN) architecture where only text and image data were combined. Therefore, based on this work, the use of both text and image data improves the detection of fake news.

The study [16] provides an overview of existing tools suitable for detecting FN and an overview of websites that can be used for fact-checking. They point out the importance of conveying techniques for the detection of disinformation and FN to the public, such as PolitiFact, a fact-checking tool.

Other works focused on more accurate approaches based on ensemble learning, where more models are learned to vote about a final decision, as described in the work [17]. The paper used strong methods of deep learning for the generation of an ensemble of models. A different approach to research was utilized in [18] via the application of a variety of ensemble strategies, with the aim of enhancing the efficiency of detection for a particular set of models, an example being the use of random forest voting for the fine-tuning of convolutional neural networks.

A different work tackled the task of fake news detection in relation to COVID-19 by employing the BERT architecture on data from social media networks. Along with the textual content of the posts, the authors also included nine additional attributes in relation to the user or the content made by the user, such as number of friends, views, or the use of hashtags in the user’s posts. The attained results showed an F1 score of approximately 85% [19]. In the case of [20], the detection of fake messages was conducted with the use of an architecture based on a CNN and LSTM. For vector representation, the authors used W2V. The dataset contained the titles and textual content of news articles accompanied by associated articles labeled as sources. The aim of this study was to assess whether the articles bore resemblance as well as relation to their respective sources. The authors achieved satisfactory results with a relatively simple architecture, with an F1 score = 0.97.

## 2. Materials and Methods

To generate models for the recognition of disinformation in the form of fake news in text, we have provided text data vectorization and subsequently learned models in the form of deep neural networks.

### 2.1. Data Description

Since it was a problem to obtain suitable text data in the Slovak language, we created a new dataset of fake and neutral, real texts. The created dataset contained 13,317 posts in the Slovak language focused on COVID-19 disease which were collected between February and October 2020. These posts were collected using the MonAnt platform. They were extracted from websites such as zemavek.sk, hlavnespravy.sk, slobodnyvysielac.sk, and badatel.net, which can be considered untrustworthy according to the web source konšpiratori.sk. Among the trustworthy sources were e.g., ta3.com or aktuality.sk. The data were annotated in a manner where each annotator had to answer two questions when annotating a given post. The first question was whether the given post contained fake news, where the annotator had a choice of answers—yes or no. In the second question, the annotator had to choose how sure he/she was of his/her answer, where he/she had the choice of the following options:

- Definitely sure;
- I am sure;
- More or less sure;
- Somewhat sure;
- Not sure at all.

Each of these answers had its own weight. For each post, the weighted sum of the values was divided by the number of different annotations. Then, according to the threshold value, false or true messages were assigned to a class. Of these 13,317 posts, 12,955 were scored as true and 362 were scored as false.

The problem was the significant imbalance between the number of fake news examples and the number of true news examples. Therefore, we decided to use only a certain fraction of the true posts, to use all the fake posts obtained from this dataset, and to supplement the fake ones. The goal was to add fake posts so that we had about a thousand of them in the dataset. To extract the fake news, we decided to use the ParseHub tool—a web application that allows to extract data from web pages using automated web scraping. A major advantage was the ability to extract data from various types of web pages, e.g., also from pages that contained dynamic content.

Before we started extracting the data, we needed to select suitable sources that may contain fake news. To avoid downloading unnecessary amounts of news that had nothing to do with the COVID-19 topic, we also selected sources based on whether the source was searchable by post category (e.g., badatel.net allows to set the search category to COVID-19 news). Furthermore, we were also guided by the ratings on konspiratori.sk, where it is possible to find information about the untrustworthiness of sources. The following are the names of selected websites that met our criteria accompanied with their rating on konspiratori.sk (the rating range is from 0 to 10, where the value 10 represents a very untrustworthy website):

- badatel.net—9.6;
- zemavek.sk—9.5;
- slovanskenoviny.sk—8.6;
- dostojneslovensko—8.5.

The actual extraction of the posts was performed with the use of the ParseHub tool. We opened the downloaded data in Excel Power Query, transformed the output, and saved it in .csv format. We then read at least 25% of each post and consequently removed posts for which our confidence that they were fake was not at the level of “I am sure”. In this way, we obtained 643 messages, which together with the previous ones created the dataset with a total 1005 fake news pieces.

We decided to select the true reports at a ratio of approximately 1 to 4, i.e., to have 4020 true reports in the final dataset. We filtered these in 2 ways:

- The first way included the condition that the minimum number of characters of the message should be 1300 (since the downloaded fake news pieces were larger in size) and the condition that the average rating for the message had to be lower than 0.1 (the rating had a range from 0.01—I am definitely sure that the message does not contain misinformation—to 0.99—I am definitely sure that the message contains misinformation).
- The second way had three conditions, namely that the number of characters was at least 1300, the average rating for the report was lower than 0.26, and that the report was annotated by at least two people.

If a message met the criteria of both ways, it was included only once. Based on these two ways, we extracted 4104 true messages into the final dataset. The data were further pre-processed using the Python programming language in the following steps:

- Removal of HTML tags;
- Removal of non-alpha-numeric characters;
- Removal of hypertext links;
- Change to lower cases;
- Tokenization;
- Deletion of stop words;
- Stemming process.

The dataset was mixed, and we set up a seed to replicate the experiments as accurately as possible. The data were divided at a ratio of 80 to 20, where 80% were data for training and 20% of the data were for model testing. Next, we converted the text into sequences of integers, which were afterwards aligned to equal lengths, called padding. We created W2V (Word2Vect) representations using the word2vec algorithm to train vector representations of words, which we later used in the neural network in the embedding layer. W2V representations are created by capturing the meaning and semantics of words based on the context in the text. The word2vec algorithm uses neural networks to learn to predict words that occur near a given word in a text. This algorithm is considered as one of the best techniques for word representation in machine learning.

### 2.2. Deep Learning of Artificial Neural Networks

With the consideration of previously conducted research, for instance, [7], we concentrated on the most acknowledged methods of deep learning, namely convolutional and recurrent networks.

***A convolutional neural network*** is one of the deep neural networks, which operates by using the mathematical function of convolution. This function can be described as a multiplication of two functions. To achieve this, the network applies convolution filters, also known as kernels. The filters are generally represented in the form of matrices, mostly of square dimensions, which are then used in convolutional layers. Another feature of CNNs are the so-called pooling layers. The aim of pooling layers is the reduction in input size. By combining the two layers, it is possible to reduce the resulting number of parameters as well as noise in comparison to a fully interconnected network. In certain cases, the reduction in resolution in every individual convolutional step could prove unsuitable. Any neural network containing at least one convolutional layer can be regarded as a convolutional network [21].

While being used also in text data processing, the initial idea for the use of CNNs was the processing of image data; however, with the use of one-dimensional convolution, it is possible to spread the reach of CNNs into the textual domain. In this case, the kernel, as the name suggests, conducts movement only in one direction while encompassing the entire length of a word vector in the other dimension. After the first convolution, we receive the result in the form of a single column. It is important to point out that this vector is shorter than the initial number of words that appear in the input sequence, which is caused by an inherent feature of the convolution—resolution reduction. This problem is generally solved with the application of the so-called “padding” in the subsequent voting layer—meaning, a zero vector is added to both the beginning and end of the input string. The described type of padding is commonly known under the name of “same”, implying that the input and output are of the same size. Other types of padding are available. Each convolutional layer contains several convolutional filters, where the values that are present within these filters represent the parameters of the convolutional layer. The user has the ability to adjust the number of filters via the hyperparameters of the network. Therefore, passing one filter through the input vectors provides us with one output vector after convolution. For each individual filter, we receive one vector. After passing all filters, the result is represented in the form of a matrix.

The following step is dimensionality reduction, which is achieved with the use of pooling layers. In the case of convolution being applied to pictures, a kernel passes over individual pixels and provides a result based on the pooling operation of a vector, where, for instance, the highest present value is taken (max pooling) or the values of the vector are averaged (average pooling). The kernel size can be adjusted by changing the values of the “pool size” hyperparameter. To adjust the movement of the kernel, a step size is set by modifying the values of a “stride” parameter, which affects the number of pixels which are taken at once in the moving direction. In the case of textual data, the pooling window moves along words selected from convolutional layers. By performing this, in several layers the number of features is reduced significantly. The result is transferred to the fully connected layer, which is then passed to the output neuron (or more of them in the case of multiclass classification) for final classification [22]. Convolutional networks are used to solve various tasks such as object recognition, video compression, speech recognition, and natural language processing [23].

***Recurrent neural networks (RNNs)*** are a type of artificial neural network often used for sequence analysis. This means that they are specifically designed to work with data that contain information with a time context. RNNs consist of several layers, and these layers are modeled sequentially so that sequences of words and relations between them can be modeled. The RNN has an excellent ability to capture contextual information from sequences of words, and this information is effectively used to achieve the data classification process [23]. This type of network has a basic function, which is to partially remember what was on the input in the past tense context. This is ensured thanks to the existence of a hidden state and recurrent connections between certain neurons or layers. As a result, for example, neurons in the hidden layer are equipped with two types of connections. In addition to being connected to another layer, each neuron also has a recursive connection to itself.

Recurrent network training may not be that simple, as there may be a problem of gradient disappearance. Training models with multiple layers can lead to gradients that are too small or too large, as errors can be transmitted through these layers. When training on longer sequences of words, this problem is accelerated, which means that training is more difficult. Recurrent networks work well on short sequences, but with longer ones, predicting the next part of the sequence may require information that was obtained almost at the beginning. This information is often lost in classical recurrent networks. Long-short term memory (LSTM) and gated recurrent units (GRUs) can address this memory problem.

**Figure 1:**
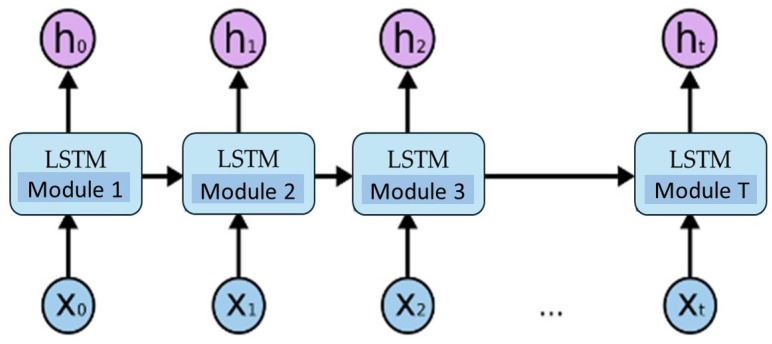
Illustration of the chain of LSTM blocks [25].

LSTM (long short-term memory) is a type of recurrent neural network that is specific in its ability to preserve information, making it a prime candidate for storing information that may otherwise get lost in a longer sequence of words. The base structure of LSTM comprises repeating modules knows as LSTM blocks, which are linked to each other and form a chain (see Figure 1). Each block contains 4 layers, and each layer includes individual parameters of the network which are trained. The layers interact mutually in a number of different ways. In individual cells, gates (input gate, forget gate, and output gate) are used to remove or add information to the state of the block. Information which is represented as a vector of words is subsequently passed through the gates, which are made from neurons that are equipped with a sigmoidal activation function. The blocks contain all three types of gates, which adjust the state in which the blocks find themselves at the current timestep. The role of a forget gate is to decide whether the passed information is to be forgotten or which part of it should be disposed of [24].

Several variations of LSTM use this principle; however, certain variations are applied to the blocks. One of the most widely known is the GRU (gated recurrent unit). It unifies the input and forget gates into a single one [25], meaning that the architecture of the GRU is simpler than LSTM’s due to it having only two gates. A GRU is simpler and performs faster when compared to LSTM; however, this results in its impaired modeling capacity. When provided with larger amounts of data, LSTM tends to achieve a better performance than a GRU [26].

## 3. Methodology

Our research was conducted in following steps:

- Splitting of the data into three parts for three clients–edges;
- Design of three different neural network architectures based on CNN, a combination of CNN and LSTM, and multiple LSTM;
- Federated learning implementation;
- Application of the federated learning to the three local datasets, using all three designed neural network architectures;
- Comparison of test results using federated learning and without federated learning.

We prepared the training dataset and testing dataset and divided the training data into three equal parts for each edge–client. Only the test dataset was used for evaluation (precision, recall, F1 metric) on the global model after each round of model training.

### 3.1. Design of CNN Model Architecture

The first model architecture was based on a convolutional neural network (CNN). The loss function was binary cross entropy, and the learning was optimized using the Adam optimizer. The number of runs was set to 10 after several experiments. The number of epochs was 1 for each model, and the batch size had a value of 25. The topology of this neural network is presented in Table 1.

The first layer of this neural network is Embedding, which represents words in text using a vector of size 150. This architecture contains several layers of a convolutional network (Conv1D) that try to identify different patterns in the text using filters of size 5. The MaxPooling1D layers are then used to reduce the dimensions after processing by previous convolutional layers and thus reduce the number of parameters in the network. To improve performance and prevent overfitting, a Dropout layer with a value of 0.3 is used in the model. Among the last layers of the architecture, GlobalMaxPooling1D is used to obtain one value for each filter. Finally, the output from this layer is used to predict a single binary output using the Dense layers with ReLU and sigmoid activation functions. Table 2 presents the testing results of the model CNN based on the topology in Table 1 without federated learning, meaning that the model was learned on all undivided data.

### 3.2. Design of CNN + LSTM Model Architecture

The second neural network architecture is based as a CNN model as well as an LSTM model. This architecture consists of the following layers:

1. An Embedding layer to transform the input text to the form of trained word embeddings via word2vec;
2. A Conv1D layer containing 32 filters with a window size of 3 and a ReLU activation function, which provides feature extraction from the input text;
3. A Dropout layer, which prevents pre-training of the model and removes some random outputs from the previous layer;
4. A MaxPooling1D layer, which reduces the output dimensions from the convolutional layer;
5. An LSTM layer, which contains 32 neurons and a Tanh activation function and allows the learning model to store the long-term dependencies between words of the input text;
6. Another Dropout layer, which removes some random outputs from the layer before;
7. A Dense layer as the second to last layer, containing 8 neurons with a ReLU activation function;
8. A Dense layer as the output layer containing 1 neuron with a sigmoid activation function, which is used for binary classification of the model output.

As a loss function, binary cross-entropy was used. We used the Adam optimization algorithm, and after several experiments, we decided on 10 rounds of model learning. Table 3 presents the topology of this neural network model.

Table 4 shows the testing results of the CNN + LSTM model based on the topology in Table 3 without federated learning, meaning that the model was trained on all undivided data.

### 3.3. Design of Multiple LSTM Model Architecture

The third neural network architecture is based on a multiple LSTM model. This topology contains the following layers:

1. An Embedding layer that transforms the input text into trained word embeddings using the word2vec algorithm;
2. An LSTM layer containing 64 neurons with the Tanh activation function, using which the model can store long-term dependencies between the words of the input text;
3. A Dropout layer, used to prevent overfitting of the model and remove some random outputs from the previous layer;
4. A second LSTM layer containing 32 neurons with the Tanh activation function, which enhances the preservation of long-term dependencies of the text words;
5. Another Dropout layer, used to remove random outputs from the previous layer;
6. A Dense layer, similar to the previous one, containing 16 neurons with a ReLU activation function;
7. A Dense layer as the last output layer, containing a single neuron with a sigmoid activation function, which is used for binary classification of the model output.

During the development of this model, we decided to use a binary cross-entropy loss function. We chose the Adam algorithm for the optimization, and after iterative testing, we decided to train the model for 10 rounds. Table 5 presents the third neural network architecture with multiple LSTM layers.

Table 6 shows the testing results of the model with multiple LSTM based on the topology in Table 5 without federated learning, meaning that the model was learned on all undivided data.

The results of the experiments presented in Table 2, Table 4 and Table 6 show that the most effective model was the third architecture of multiple LSTM layers and the least effective was the first CNN architecture when the training was conducted on the entire undivided dataset without the application of federated learning. Experiments with federated learning applied are described in the following section.

## 4. Federated Learning

Modern distributed networks and devices generate large amounts of different data on daily basis. With their ever-increasing power and capabilities, users may have concerns related to the transmission of private data and information. For example, as the data storage capabilities and computing capacity of devices in distributed networks multiply, it is not a problem to use the local storage capacity at each local node; thus, protecting privacy when transferring sensitive data is not necessary because user-generated data remain in local devices. Therefore, the recent preference for local data storage and training models locally prefers federated learning, which allows for training models directly on remote devices. Federated learning is a possible solution because it can achieve a reduction in data leakage [27]. The development of this technology has the potential to transform current smart IoT systems with advanced federated learning architectures into privacy-enhancing systems. Federated learning [28] is a form of distributed learning of models. Instead of learning on a central endpoint, training is performed on multiple clients before aggregation. Federated learning has been used in many different devices, for example, medical image classification and other structured data to IoT devices settings.

We classify federated learning into two types based on the networking structure [29]:

- Centralized federated learning—the most widely used approach to federated learning. The approach is based on a central server and a set of clients. In each training cycle, all clients provide parallel training of the neural network models using their locally obtained datasets. Consequently, all clients transfer the sets of models’ parameters to the central server for aggregation using, for example, a weighted averaging algorithm. In the central server, a global model is created and sent to clients to re-train the aggregated model again on their local data. Finally, when the process of training is finished, each client has at its disposal the same global model and a personalized version of its local model. Here, the central server is considered a key component of this kind of federated learning to centrally aggregate local models and distribute the global model to the clients.
- Decentralized federated learning—a network topology, opposed to centralized federated learning, where all clients communicate in a peer-to-peer manner. Thus, clients train local models on their datasets in each communication round. Afterwards, each client updates the models received from neighboring clients via peer-to-peer communication to reach a consensus on a global update. Decentralized federated learning is designed to replace the central server fully or partially if, for example, a communication with the server is unavailable.

Federated learning increases the privacy of the data by training machine learning models on local premises where the data reside, so no data transfer is required. It then allows only the resulting model to be shared. Therefore, this learning avoids the need to share data between local stations—clients [30].

Parallel federated learning is a type of centralized federated learning of models in a distributed environment. For this technique, local neural network models are trained on each client–edge model in the first step. Then, the weights obtained from the local models are averaged and tested on the global model in an aggregator. Then, the weights obtained from the global model are returned to the local models, and the neural network is trained there further again. This cycle is repeated until the specified number of training rounds reached.

### 4.1. Federated Learning Implementation

**Figure 2:**
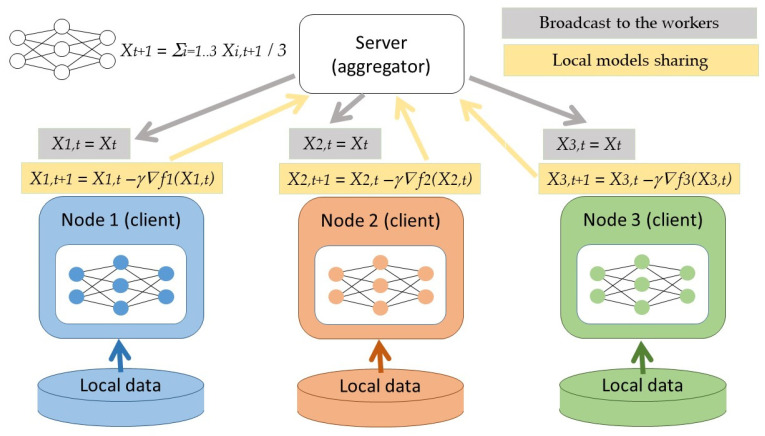
Architectural diagram of the parallel federated learning with three edges–clients.

We used parallel federated learning and applied it through the Ray library. In our solution, we simulated federated learning using Ray as a framework for distributed task processing and created a decentralized network of clients on a single machine, where each client had its own data. At the beginning of the method, it was necessary to determine the number of rounds for training, that is, how many times to apply the full cycle of federated learning. Figure 2 shows the parallel federated learning cycle with three edges–clients.

As we can see in Figure 2, the server and all clients share the same model structure—one of the above-mentioned model architectures (CNN, CNN + LSTM, or multiple LSTM). The server initializes its model copy (global model) and starts a certain number of learning rounds. All rounds are conducted with the same structure. In a round starting at time t, the server shares its global model (i.e., weight vector X_t_) with the clients. Each client uses the weights to initialize its local copy of the model (X_i,t_ = X_t_) and performs one learning epoch utilizing its locally available data (using an algorithm based on gradient descent) to produce a local model. Subsequently, the clients share their local models (i.e., weight vectors X_i,t+1_) with the server. Finally, the server aggregates the local models in the form of a new global model X_t+1_.

### 4.2. Applying Federated Learning to Three Local Datasets, Using the Designed Topologies

The results of the experiments with federated learning on the first deep learning model with the CNN architecture are presented in Table 7. Three models of CNNs were trained for three clients and were subsequently aggregated in the central server.

Table 8 presents the results of the final CNN model after all cycles of parallel federated learning with the topology presented in Table 1. The final model is the result of aggregation in the central server.

The next two tables present the results of aggregated models trained using parallel federated learning. Table 9 shows the effectiveness of the CNN + LSTM aggregated model and Table 10 shows the effectiveness of the multiple LSTM aggregated model.

The results of the experiments presented in Table 8, Table 9 and Table 10 show the opposite trend to the results provided on the whole undivided dataset without applying federated learning. Using federated learning, the most effective model was the first model with the CNN architecture and the least effective was the third architecture based on multiple LSTM layers, although the differences in F1 rate, precision, and recall values are negligible.

## 5. Discussion

Generally, federated learning represents a multifaceted topic. This paper is focused on one facet only—learned model efficacy with and without federated learning. In addition to the model efficacy, there are other challenges that the users of federated learning face in different contexts. Let us consider a potential scenario in the context of fake news detection. In this scenario, users are able to mark news they just read (in their favorite content sources such as blogs, discussions, news portals, etc.) as real/fake news and, in this way, to build their own stock of labeled news examples. Such a potential federated network could face the following challenges:

- Communication complexity;
- Data heterogeneity;
- Device heterogeneity;
- Privacy.

*Communication complexity*—The network can consist of a substantial number of devices acting as clients (e.g., smart phones). As a result, massive communication between these devices and the server aggregating their messages can become an issue. Possible strategies to decrease the amount of communicated data can be based on more autonomous local computing (devices perform more learning steps before sharing back their local models) or more effective data transfer (using compression techniques or sharing parts of their local models only).

*Data heterogeneity*—News pieces from content sources consumed by different users are typically heterogeneous, varying in the size of collected data, news length, used language, and topic focus. Thus, data collected by different devices cannot be considered i.i.d. (independent and identically distributed), which represents a challenge for aggregating shared local models. Possible strategies are multi-task learning (learning separated but related models) or considering the relationship between devices and their associated distributions.

*Device heterogeneity*—User devices differ in computational power due to variability in hardware (ranging from smart phones to tablets to computers), network connectivity (3–5G, Wi-Fi), availability (only a small fraction of devices are active simultaneously), and reliability (an active device can drop from the network for a time period). Possible strategies in this case are based on asynchronous communication between the client and server, enabling partial participation of devices in the federated network, or on active sampling (active selection of participating devices for a specific round).

*Privacy*—Even if users are willing to contribute to building models for fake news detection, they do not want to expose information on which news they consume from which data sources. This information cannot leak from their devices (through identifying sensitive text patterns from model weights), neither to a third party nor an aggregation server (the server could be untrustworthy)—a major concern of federated learning. Possible strategies are based on differential privacy or secure multi-party computation.

## 6. Conclusions

The results of federated learning were as follows. The best precision for fake news was achieved by the third model with multiple LSTM layers, but it simultaneously achieved the worst recall for fake news with a value of 0.85. For recall for fake news, the CNN model performed the best with a value of 0.93. As for the F1 score metric, the first CNN model was also the best with a value of 0.90. The accuracy metric was 0.96 for the first CNN model, 0.95 for the second model (CNN + LSTM), and 0.949 for the third model. However, we can say that the results of the models using federated learning were very balanced and showed only slightly better results for the first and second models.

A comparison of the effectiveness of the models using federated learning and the models without federated learning showed the following. For the first architecture (CNN), the accuracy result was slightly better in the model implementing federated learning, with a success rate of 0.96 compared to the success rate of the other models of 0.95. The models without federated learning achieved almost the same but slightly increased accuracy values than the models with federated learning. It should also be noted that the parameters were tuned for the models with federated learning implemented—we only varied the batch size for the classical neural network model. We can conclude that the models with and without federated learning achieved comparable results. This leads to the important conclusion that sensitivity-aware federated learning does not degrade the efficiency of the models, even though they are learned on smaller local datasets.

This paper offers an approach to generate models able to detect fake news related to the COVID-19 pandemic in the Slovak language. The best achieved accuracy was 0.96, with the highest recall value of 0.93.

Based on our experience, we recommend the use of federated learning, which brings many benefits over classical training of machine learning models. Especially, for example, for medical data, where data security is of high importance. Training can take place on local data at individual stations or edges.

In the future, we will also research the applicability of our models in the Slovak language, where we will develop this area in two directions. First, we will collect, merge, modify, and integrate datasets in the Slovak language [31]. Secondly, we will apply transformers to models trained by deep learning on English data. Our experiences in the detection of fake news about COVID-19 could be helpful in early recognition of the speaker’s age, e.g., to find when adults are pretending to be a teenager to influence children’s chat groups [32]. In this case, we would use multimodal data, including text and speech, in the Slovak language.

## Figures and Tables

**Table 1:** Topology of the CNN model trained using deep learning. Total parameters number is equal to number of trainable parameters—8,604,153 (number of non-trainable parameters is 0).

Layers	Output Shape	Parameters
Embedding	(None, 3152, 150)	embedding_11, 8333400
Conv1D	(None, 3148, 64)	conv1d_33, 5, 48064
MaxPooling1D	(None, 787, 64)	maxpooling1d_22, 0
Dropout	(None, 787, 64)	dropout_22, 0.3
Conv1D	(None, 783, 128)	conv1d_34, 5, 41088
MaxPooling1D	(None, 195, 128)	maxpooling1d_23, 0
Dropout	(None, 787, 64)	dropout_23, 0.3
Conv1D	(None, 191, 256)	conv1d_35, 5, 164096
GlobalMaxPooling1D	(None, 256)	global_max_pooling1d_11
Dense	(None, 64)	dense_33, ReLU
Dense	(None, 16)	dense_34, ReLU
Dense	(None, 1)	dense_35, Sigmoid

**Table 2:** Effectiveness of CNN model with the topology presented in the Table 1, without applying federated learning.

CNN	Precision	Recall	F1 Rate	Support
Real_news	0.97	0.97	0.97	814
Fake_news	0.89	0.88	0.89	208
Accuracy			0.95	1022
Macro average	0.93	0.93	0.93	1022
Weighted average	0.95	0.95	0.95	1022

**Table 3:** Topology of the CNN + LSTM model trained using deep learning. Total parameters number is equal to number of trainable parameters—8,356,425 (number of non-trainable parameters is 0).

Layers	Output Shape	Parameters
Embedding	(None, 3152, 150)	embedding_1, 8333400
Conv1D	(None, 3150, 32)	conv1d, 14432
Dropout	(None, 3150, 32)	dropout_2, 0.3
MaxPooling1D	(None, 1575, 32)	maxpooling1d, 0
LSTM	(None, 32)	lstm_1, 8320
Dropout	(None, 32)	dropout_3, 0.3
Dense	(None, 8)	dense_3, ReLU, 264
Dense	(None, 1)	dense_4, Sigmoid, 9

**Table 4:** Effectiveness of CNN + LSTM model without applying federated learning.

CNN + LSTM	Precision	Recall	F1 Rate	Support
Real_news	0.96	0.98	0.97	814
Fake_news	0.91	0.86	0.88	208
Accuracy			0.96	1022
Macro average	0.94	0.92	0.93	1022
Weighted average	0.96	0.96	0.96	1022

**Table 5:** Architecture of the multiple LSTM model trained using deep learning. Number of total parameters is equal to number of trainable parameters—8,401,401 (number of non-trainable parameters is 0).

Layers	Output Shape	Parameters
Embedding	(None, 3152, 150)	embedding, 8333400
LSTM	(None, 3152, 64)	lstm, 55040
Dropout	(None, 3152, 64)	dropout, 0.3
LSTM	(None, 32)	lstm_1, 12416
Dropout	(None, 32)	dropout_1, 0.3
Dense	(None, 16)	dense, ReLU, 528
Dense	(None, 1)	dense_1, Sigmoid, 17

**Table 6:** Effectiveness of multiple LSTM model without applying federated learning.

LSTM + LSTM	Precision	Recall	F1 Rate	Support
Real_news	0.97	0.99	0.98	814
Fake_news	0.92	0.85	0.88	208
Accuracy			0.97	1022
Macro average	0.95	0.93	0.94	1022
Weighted average	0.96	0.96	0.96	1022

**Table 7:** Effectiveness of federated learning of CNN models trained on three local datasets on three clients’ local sides. The topology of the model is presented in Table 1.

CNN	Precision	Recall	F1 Rate	Support
	**Client 1**			
Real_news	0.99	0.93	0.96	814
Fake_news	0.77	0.96	0.86	208
Accuracy			0.93	1022
Macro average	0.88	0.94	0.91	1022
Weighted average	0.95	0.93	0.94	1022
	**Client 2**			
Real_news	0.98	0.97	0.97	814
Fake_news	0.89	0.91	0.90	208
Accuracy			0.96	1022
Macro average	0.93	0.94	0.93	1022
Weighted average	0.96	0.96	0.96	1022
	**Client 3**			
Real_news	0.95	0.99	0.97	814
Fake_news	0.95	0.78	0.86	208
Accuracy			0.95	1022
Macro average	0.95	0.88	0.91	1022
Weighted average	0.95	0.95	0.95	1022

**Table 8:** Effectiveness of final aggregated CNN model trained using parallel federated learning with the topology presented in Table 1.

CNN	Precision	Recall	F1 Rate	Support
Real_news	0.98	0.97	0.97	814
Fake_news	0.88	0.93	0.90	208
Accuracy			0.96	1022
Macro average	0.93	0.95	0.94	1022
Weighted average	0.96	0.96	0.96	1022

**Table 9:** Effectiveness of aggregated CNN + LSTM model trained using parallel federated learning with the topology presented in Table 3.

CNN	Precision	Recall	F1-Rate	Support
Real_news	0.97	0.97	0.97	814
Fake_news	0.88	0.89	0.89	208
Accuracy			0.95	1022
Macro average	0.93	0.93	0.93	1022
Weighted average	0.95	0.95	0.95	1022

**Table 10:** Effectiveness of aggregated multiple LSTM model trained using parallel federated learning. Table 5 contains the topology of this model.

CNN	Precision	Recall	F1-Rate	Support
Real_news	0.96	0.97	0.97	814
Fake_news	0.89	0.85	0.87	208
Accuracy			0.95	1022
Macro average	0.93	0.91	0.92	1022
Weighted average	0.95	0.95	0.95	1022

## Data Availability

Datasets containing 13,317 posts in the Slovak language focused on COVID-19 disease titled “fake_news.csv” and “real_news.csv” are available at (https://kristina.machova.website.tuke.sk/useful/) (accessed on 20 May 2024).
